# Interactive segmentation with curve-based template deformation for spatiotemporal computed tomography of swallowing motion

**DOI:** 10.1371/journal.pone.0309379

**Published:** 2024-10-21

**Authors:** Yuki Kimura, Takashi Ijiri, Yoko Inamoto, Takuya Hashimoto, Yukihiro Michiwaki

**Affiliations:** 1 Shibaura Institute of Technology, Koto-ku, Japan; 2 Fujita Health University, Toyoake-shi, Japan; 3 Tokyo University of Science, Katsushika-ku, Japan; 4 Toho University, Ota-ku, Japan; University of the Pacific Arthur A Dugoni School of Dentistry, UNITED STATES OF AMERICA

## Abstract

Repeating X-ray computed tomography (CT) measurements over a short period of time allows for obtaining a spatiotemporal four-dimensional (4D) volume image. This study presents an interactive method for segmenting a 4DCT image by fitting a template model to a target organ. The template consists of a three-dimensional (3D) mesh model and free-form-deformation (FFD) cage enclosing the mesh. The user deforms the template by placing multiple curve constraints that specify the boundary shape of the template in 3D space. We also present curve constraints shared over all time frames and interpolated along the time axis to facilitate efficient curve specification. Our method formulates the template deformation using the FFD cage modification, allowing the user to switch between our curve-based method and traditional FFD at any time. To illustrate the feasibility of our method, we show segmentation results in which we could accurately segment three organs from a 4DCT image capturing a swallowing motion. To evaluate the usability of our method, we conducted a user study comparing our curve-based method with the cage-based FFD. We found that the participants finished segmentation in approximately 20% interaction time periods on average with our method.

## Introduction

X-ray computed tomography (CT) is a technique used to obtain a volumetric image of the interior of a solid object. Repeating CT measurements over a short time period produces a spatiotemporal four-dimensional (4D) volume image. This 4DCT image is used to analyze the dynamic motion and function of the human body, such as the heart [1], lung [2], and ankle joint [3]. Specifically, this study focuses on swallowing, a motion that transfers food from the oral cavity to the stomach. Because deterioration of the swallowing function can lead to serious health problems, such as aspiration pneumonia, swallowing is an important target for analysis. Whereas swallowing has been commonly studied using videofluorography images [4, 5], some researchers have applied 4DCT for swallowing analysis [6–9]. However, a tool to efficiently segment specific organs from 4DCT images is needed for these applications.

Medical volume segmentation is an active research field, and many fully automatic methods have been proposed utilizing template registration [10–12] or deep learning [13, 14]. However, fully automatic methods suffer from the difficulty present in making minor corrections when the results contain errors. In addition, because swallowing 4DCT images are not commonly captured, it is difficult to prepare a sufficient training dataset. Other researchers have invented interactive three-dimensional (3D) volume segmentation tools allowing the user to roughly specify inside and outside voxels [15, 16] or place contour constraints [17, 18]. However, in a swallowing 4DCT image, the boundaries of fast-moving organs blur, and achieving accurate results only from rough inside/outside constraints is difficult. In addition, adapting these methods to 4DCT is not straightforward, as it requires work on a huge amount of frames.

The goal of this study is to present a tool for efficiently segmenting organs related to swallowing from 4DCT images. Unlike a standard CT image that captures static organs, in a swallowing 4DCT volume, the boundaries of quickly moving organs are blurry. Therefore, a tool that allows the user to carefully observe the CT and specify the shape of the target organ is desirable. We introduce an interactive segmentation tool, in which the user deforms a template model by placing curve constraints in 3D space. We also provide shared curve constraints to support efficient curve specification in 4DCT; the shared curve constraints are duplicated in all the frames, with its shape automatically interpolated along time.

**Fig 1:**
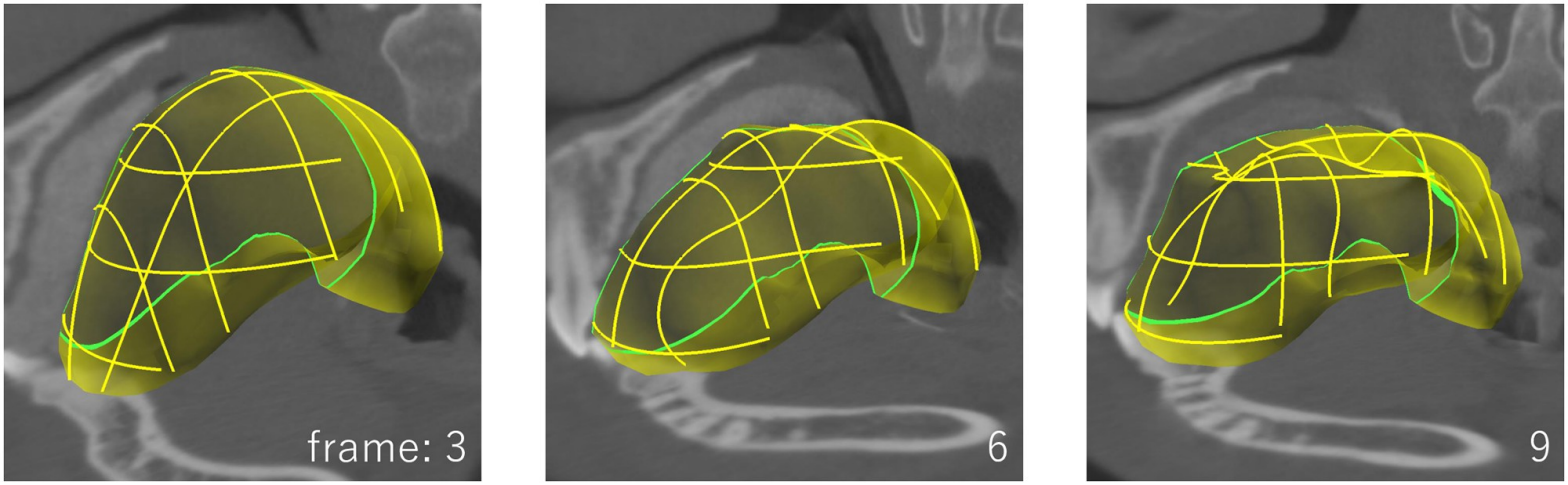
Example of tongue segmentation using a 4DCT image that captures the swallowing motion. The user specifies curve constraints at the boundary of the target organ on the cross-sectional planes of the 4DCT. Our method deforms the template fitting to the curves.

To demonstrate the effectiveness of our tool, we segmented a 4DCT image capturing the swallowing motion to extract three organs strongly involved in the swallowing process, namely, the tongue, soft palate, and larynx. Fig 1 shows representative frames of the tongue segmentation results. The segmentation of the tongue required approximately 137 minutes to complete. Because the 4DCT contains 29 time frames, the average interaction time for each frame was less than 5 min. We conducted a user study that compares our method with traditional cage-based free-form deformation (FFD) [19]. The participants achieved segmentation using our curve-based method in approximately 20% of the work time compared to the cage-based method. Because our tool allows the user to deform a template model by placing curve constraints directly on cross-sections of the CT image, time-varying shapes can be segmented quickly and efficiently.

The experiments in this paper was conducted with the approval of the Ethics Committee of Shibaura Institute of Technology (No. 22–007).

## Related work

Medical volume segmentation is an active research field, encouraging much study. In this section, we survey fully automatic and interactive segmentation methods closely related to this study.

### Fully automatic segmentation

Template-based methods involve the preparation of a template model for a target organ, deforming it to fit the target region of interest (ROI) well. Montagnat et al. deformed an elastic model to fit a volume image [10], and Pizer et al. introduced M-reps, which is a deformable model represented by small number of parameters [11]. Ecabert et al. progressively increased the degrees of freedom of a deformation model to extract multiple chambers of a heart [12]. However, our targets, i.e., organs in swallowing 4DCT, often contain unclear boundaries, complicating the ability to automatically fit templates to such ROIs. Instead, we provide an interactive tool that allows experts to check and deform template models.

Many researchers have applied deep neural networks (DNNs) for medical volume segmentation. Fully convolutional neural network [20] and U-Net [21] architectures have provided examples for semantic segmentation. Similar architectures have been introduced to segment medical 3D volumes [22, 23] and 4D volumes [24, 25]. Some studies [26, 27] have performed data augmentation for training networks using deformable models. We refer to survey papers [13, 14] for a comprehensive treatment of these studies. While DNN-based methods achieve accurate segmentation, applying them to 4DCT without a sufficient dataset would be difficult. This study aims to present a tool for the manual segmentation of swallowing 4DCT. Applying the segmentation results to DNN training is our plan for future work.

### Interactive segmentation

Researchers have presented interactive segmentation methods that allow the user to roughly specify the inside and outside of ROI through scribble inputs. Traditional region-growing methods are considered in this group. Such a method gradually grows ROI(s) from user-specified seed voxels. This method can be extended to grow multiple regions [28] or compute the growth by cellular automaton [29]. This group also includes graph cut segmentation. Given the user-specified constraint voxels (foreground/background or multi-labels), the method computes ROI(s) by constructing a flow network and finding its minimum cut [15, 16]. This method was extended to allow the user to draw constraints directly on a rendering screen [30]. In addition, Igarashi et al. presented a paint-based interface for specifying locally varying thresholds [31]. However, segmenting ROIs with ambiguous boundaries is difficult with these methods because the user can only apply rough inside/outside or threshold specifications.

Methods that allow the users to specify ROI boundaries by applying curve constraints have been presented. The key idea presented in these methods is the reconstruction of 3D shapes from user-specified constraint curves. Ju et al. [32] and Liu et al. [33] explicitly reconstructed 3D mesh models from parallel and non-parallel curves, respectively. They initialized 3D mesh models by spatial partitioning and performed smoothing. Ijiri et al. [34] used contour curves as a handle to deform the shape of the ROI. In addition, Heckel et al. [17] implicitly reconstructed 3D shapes using radial basis function interpolation. Ijiri et al. [18] extended this implicit method to the bilateral domain to fit 3D shapes to image edges. While these methods produced useful tools and accurate segmentation, they only deal with 3D volumes; thus, extending them to 4DCT is not straightforward.

## Methods

### Overview of curve-based 4DCT segmentation

**Fig 2:**
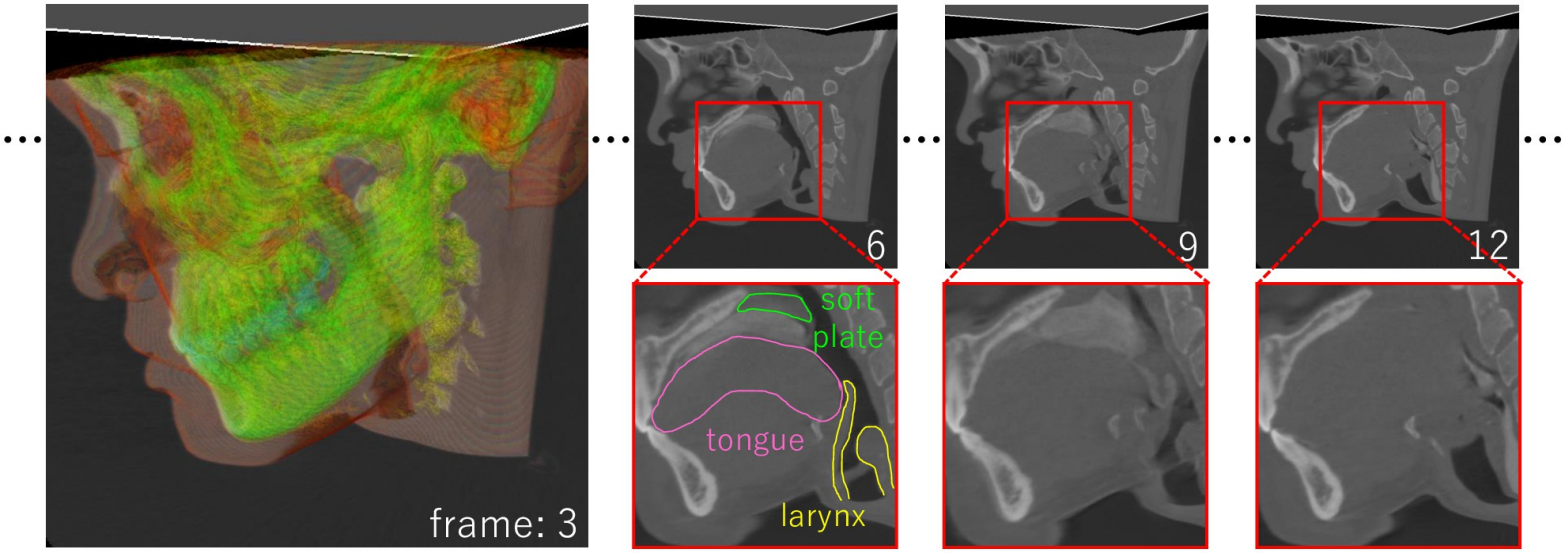
Representative frames of a swallowing 4DCT image. During swallowing, organs move quickly causing their boundaries to become blurry, especially when bolus (masticated food) passes through the throat.

The goal of this study is to propose an efficient segmentation tool for 4DCT images. Specifically, we focus on swallowing 4DCT and attempt to segment organs strongly related to the swallowing motion, namely, the tongue, soft palate, and larynx. Because the boundaries of quickly moving organs often appear ambiguous, even nearly invisible in some frames (Fig 2), we present a curve-based template registration method. The user prepares a template model for a target organ and deforms it by placing curve constraints directly on the boundary of the target. As the standard curve constraints are placed independently at each frame, we present a *shared curve* to handle time-varying shapes efficiently. The shared curve is shared with all frames, and its shape can be modified by the user in any frame and is automatically interpolated at all frames without user specification. Because the user can specify the template shape directly by applying curve constraints while observing the overall template shape and CT images, accurate and efficient segmentation is achieved, even for regions with ambiguous boundaries.

**Fig 3:**
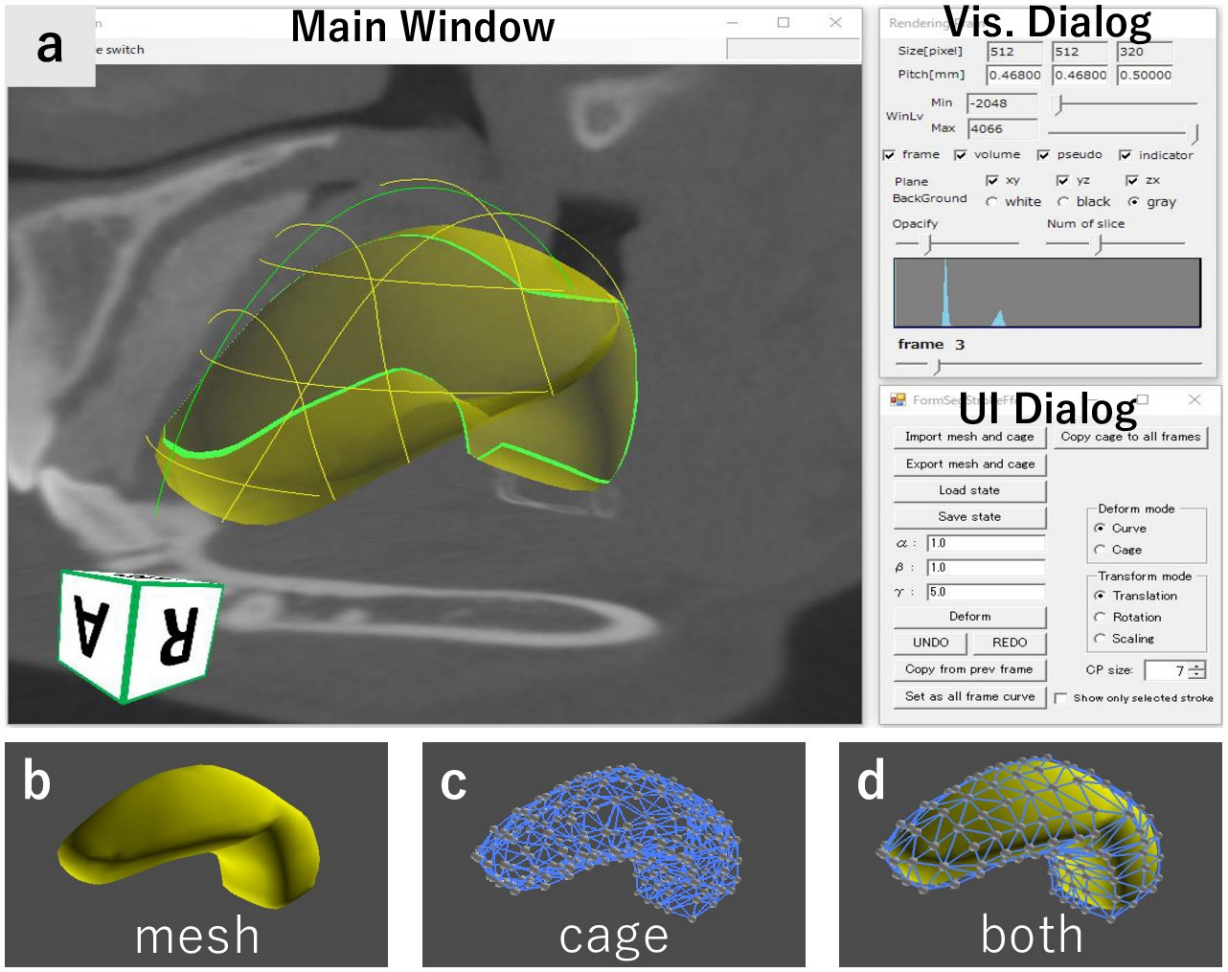
Screenshot of our 4DCT segmentation system (a) and a template model for the tongue (b-d).

Our tool is implemented as a standard application consisting of a main window, visualization dialog, and UI dialog (Fig 3a). The main window is used to observe the 4DCT image, specify curve constraints, and check segmentation results. The visualization dialog is used to modify the visualization parameters, and it contains a seek bar to navigate the time frame of 4DCT forward and backward. The UI dialog is used to manipulate the segmentation tools. The application and source codes are available online, https://doi.org/10.6084/m9.figshare.26639935.

The inputs of our method are a 4DCT volume image and template model for a target organ. Fig 2 shows an example of a swallowing 4DCT image taken with a 320-slice CT device at 0.1 seconds per frame (10 fps). Its total size is 512 × 512 × 320(3*D*) × 29(*frames*), and a 4DCT image of similar size is assumed as the input. Our template model is represented with a deformable watertight mesh model and a cage enclosing the mesh (Fig 3b–3d). The cage serves as a handle to deform the mesh through the FFD technique. We utilize harmonic coordinates [19] to associate the mesh vertices with the coordinate system defined by the cage. Our tool deforms the template mesh by transforming the cage such that the mesh fits the user-specified curve constraints. By using this FFD representation, the user can switch between two modes: our curve-based deformation mode, which supports direct and efficient specification, and the traditional FFD mode, which is indirect and inefficient but allows for detailed adjustment.

### User interface

**Fig 4:**
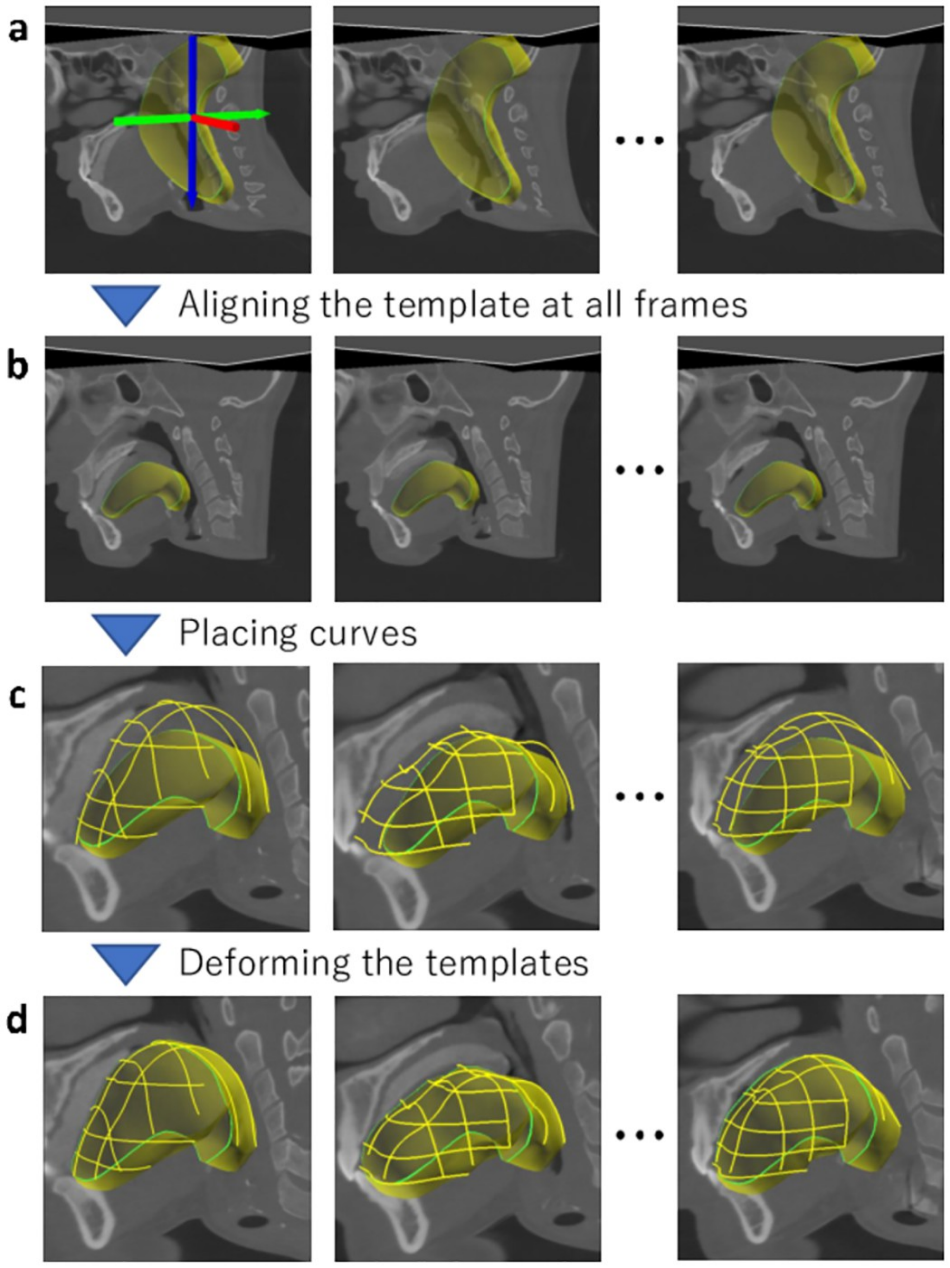
Typical segmentation process. Given a 4DCT image and a template (a), the user roughly aligns the template at a frame and copies the alignment information to all frames (b). The user then places curve constraints for all frames (c) to deform the template (d).

We depict a typical segmentation process in Fig 4 (See also S1 Video). After loading a 4DCT image and a template model, initially, the user roughly aligns the template with the target organ in 4DCT by performing translation, rotation, and scaling (Fig 4b). Next, the user places curve constraints on the boundary of the target organ at each frame (Fig 4c). When the user presses the *Deform* button, the template is automatically deformed to fit the curve constraints (Fig 4d). The user can also convert an already placed curve into a shared curve, which is duplicated in all frames and interpolated over time. If necessary, the user is able to switch to FFD mode and deform the template by dragging the cage vertices. The user can repeat the curve-based deformation until satisfied with the template shape.

#### Alignment

At the beginning of the segmentation, the user roughly aligns the template through translation, rotation, and scaling. When the user selects the translation, rotation, or scaling modes from the UI dialog buttons, the tool shows a manipulation handle in the main window (Fig 4a left), which can be dragged with a mouse. The user can copy the alignment of a frame to the other frames or manually specify the alignment for each frame.

#### Curve constraint placement

**Fig 5:**
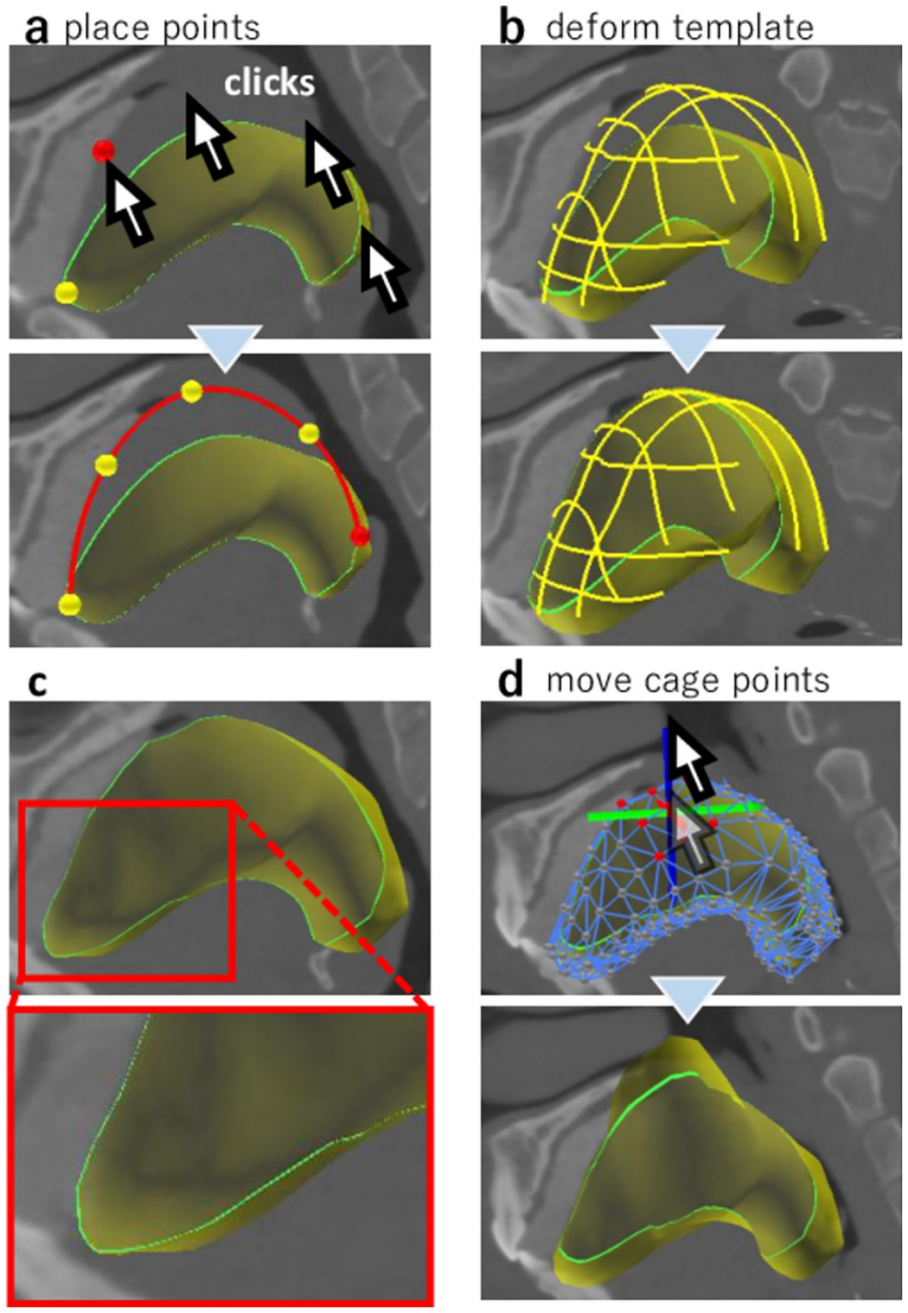
User interface. (a) The user clicks on a cross-section to add control points for a curve. (b) The system deforms the template fitting to the user-specified curves. (d) The user can also manipulate the FFD cage to deform the template.

The main task of the segmentation is to place curve constraints on the boundary of the target organs. The user first moves one of three orthogonal cross-sections (XY-, YZ-, or ZX-planes) to a position where the target organ is clearly visible. The user then clicks the cross-section to place a control point. When three or more control points are placed, our tool smoothly interpolates them with a smooth curve (Fig 5a); we used the *κ*–curve [35]. If the user clicks on a background, the curve under creation is inactivated. In this condition, the user can add a new curve by placing a new control point. The user can repeat this process to place multiple curve constraints.

Editing existing curves is also possible. If the user clicks on an existing control point, the curve that owns the point is activated (highlighted in red). Once a curve is activated, the user can move its control points by dragging them, delete control points by right-clicking on them, and add new control points by clicking on the cross-section.

When the user presses the *Deform* button in the UI dialog, our tool automatically deforms the template such that its boundary fits the specified curves (Fig 5b). This deformation is achieved by modifying the template cage. Our tool highlights the intersection between the template and cross-sectional planes with green curves, enabling the user to check the deformation results (Fig 5c). Our system allows the user to toggle between translucent and opaque visualizations of the template.

#### Curve constraint shared with all frames

In medical 4DCT, organs may move and change their shapes with the frames, but their topology are usually constant. To efficiently specify curve constraints related to such organs, our tool provides a shared curve; this curve is duplicated across all the frames, and its shape is interpolated over time.

**Fig 6:**
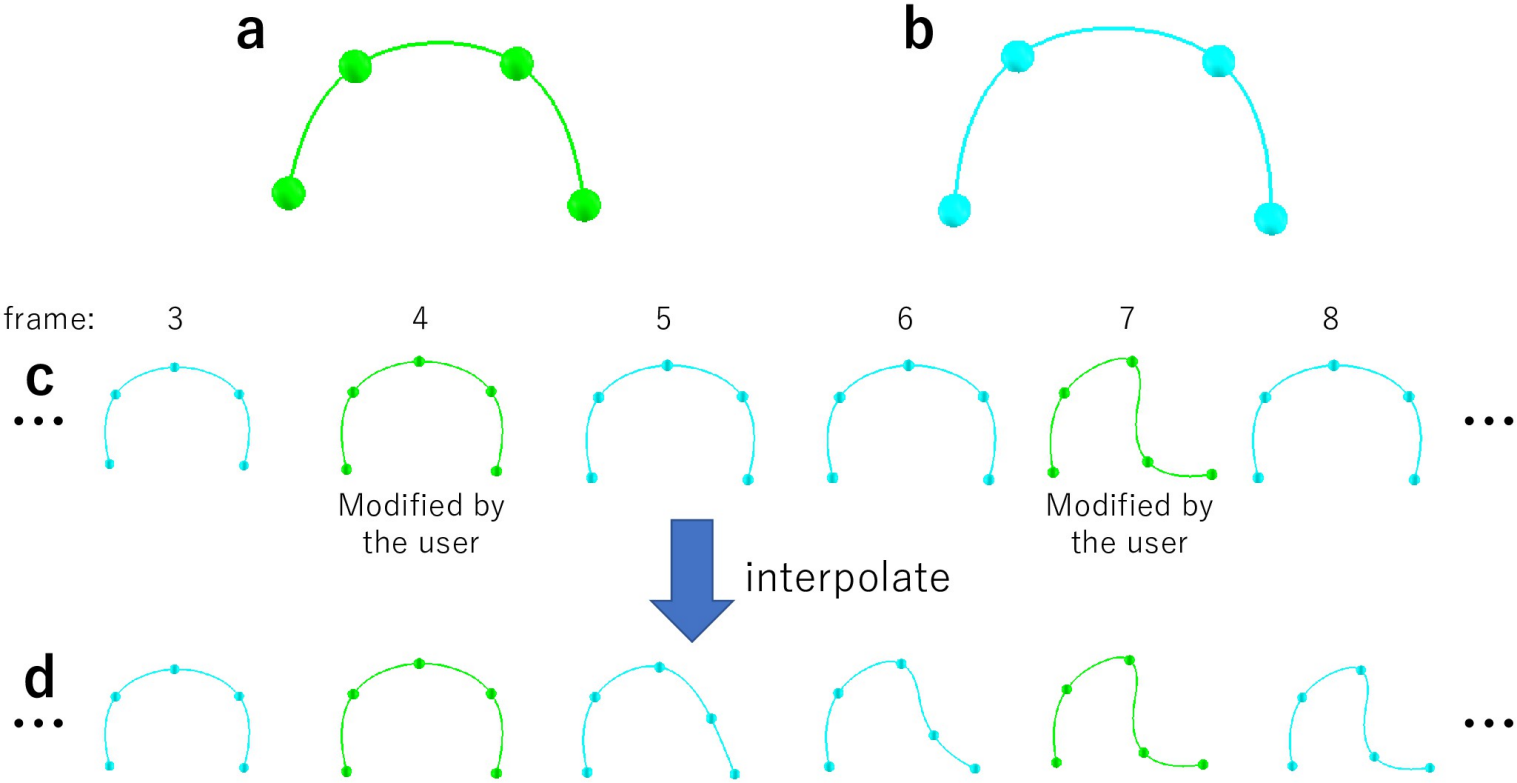
Shared curve. A shared curve is highlighted in green in the frames with user specification and in light blue in the frames without user specification. The shapes of the light blue curves are linearly interpolated along time.

When the user activates a curve constraint and presses the *set as all frame curve* button in the UI dialog, the curve is converted into a shared curve, and its copies are placed in all frames. The user can modify the shape of the curve in any frame by dragging its control points. The shared curve is highlighted in green in the frames with user modification. In the frames without user modification, the shared curve is highlighted in light blue, and its shape is linearly interpolated by using the frames with user modification (Fig 6).

#### Cage-based free-form deformation

Our tool allows the user to modify the template using traditional FFD. The tool shows the cage vertices when the user switches to FFD mode by pressing the *Cage* button in the UI dialog. The user can select and move the cage vertices via a mouse. The template mesh deforms with the deformation of the cage (Fig 5d). In 4DCT, certain organs may change their shapes substantially, making it difficult to perform initial alignment through only translation, rotation, and scaling. The FFD tool is useful for such organs. For instance, we used this tool in this study to perform the initial rough alignment of the rotational motion of the epiglottis. This tool is also available for the final fine-tuning of the template.

### Algorithm for deformation

This section describes a method to deform the template fitting to the user-specified curves. We compute the deformation independently, frame by frame. The template is represented by the mesh model M and the FFD cage C. We associate a mesh vertex with the FFD cage by using harmonic coordinates [19]; each mesh vertex **v**_*i*_ ∈ *R*^3^(*i* = 1, 2, …, *N*_*m*_) is represented by a weighted sum of the cage vertices **c**_*j*_ ∈ *R*^3^(*j* = 1, 2, …, *N*_*c*_);

$$\begin{array}{c} {\text{v}}_{i}=\sum_{j=0}^{N_{c}}a_{j}^{i}{\text{c}}_{j} \end{array}$$ (1)

where $a_{j}^{i}$ is the weight of the harmonic coordinate. We optimize the cage shape **c**_*j*_ to deform the mesh **v**_*i*_ indirectly.

**Fig 7:**
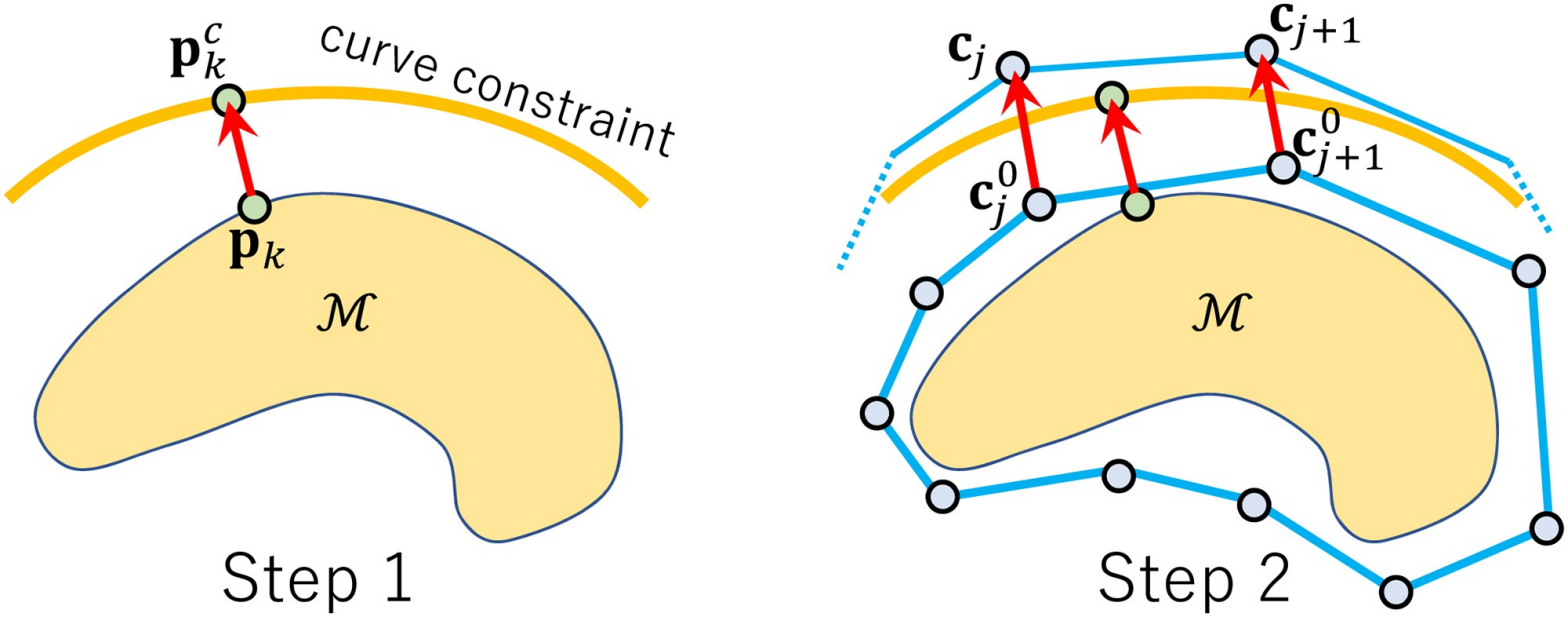
Two-step algorithm for updating the template.

Given the curve constraints, we resample all of them at an equal interval to obtain the constraint points ${\text{p}}_{k}^{c}\in R^{3},(k=1,2,\ldots ,N_{p}$), where *N*_*p*_ is the number of resampled points. We then optimize the cage shape by repeating the following two steps alternately (Fig 7):

**Step 1**: For each constraint point ${\text{p}}_{k}^{c}$, we search the point **p**_*k*_ on M closest to ${\text{p}}_{k}^{c}$. This point can be represented by the barycentric coordinate as,
  $$\begin{array}{c} {\text{p}}_{k}=\alpha_{k}^{1}{\text{v}}_{k}^{1}+\alpha_{k}^{2}{\text{v}}_{k}^{2}+\alpha_{k}^{3}{\text{v}}_{k}^{3}, \end{array}$$ (2)
  where $\left({\text{v}}_{k}^{1},{\text{v}}_{k}^{2},{\text{v}}_{k}^{3}\right)$ is a triangle of M on which **p**_*k*_ is located.
**Step 2**: We update the cage vertices **c**_*i*_ by optimizing the following cost function:
  $$\begin{array}{c} \underset{{\text{c}}_{j}}{\text{min}}\;\alpha \sum_{k=1}^{N_{p}}\left|\right|{\text{p}}_{k}-{\text{p}}_{k}^{c}{\left|\right|}^{2}+\beta \sum_{j=1}^{N_{c}}\left|\right|{\text{c}}_{j}-{\text{c}}_{j}^{0}{\left|\right|}^{2}+\gamma \sum_{j=1}^{N_{c}}||L\left({\text{c}}_{j}\right)-L\left({\text{c}}_{j}^{0}\right){\left|\right|}^{2}, \end{array}$$ (3)
  where L(·) is the graph Laplacian operator, ${\text{c}}_{j}^{0}\in R^{3}$ are the cage vertices just before this optimization, and (*α*, *β*, *γ*) are the weighting coefficients. The first term fits the mesh to the constraint points. The second and third terms maintain the cage shape in each iteration. With Eqs (1) and (2), the constraint points on the mesh **p**_*k*_ can be represented by a linear combination of **c**_*j*_. This quadratic optimization can be computed by solving a linear system about **c**_*j*_.

In our implementation, we empirically set *α* = 1, *β* = 1, and *γ* = 5 and iterate the two steps 20 times. These parameters experimentally worked well. Algorithm 1 provides a pseudo-code of our method.

**Algorithm 1** Deformation Algorithm

**Input**: Mesh model M, FFD cage C, curve constraints, parameters *α*, *β*, *γ*, *n*

**Output**: deformed mesh and cage

 1: Associate mesh vertices **v**_*i*_ to cage vertices **c**_*j*_ Eq (1) using harmonic coordinates

 2: Resample curve constraints to obtain constraint points ${\text{p}}_{k}^{c}$

 3: **for** *t* = 1 to *n* **do**

 4:  **for** each constraint point ${\text{p}}_{k}^{c}$ **do**

 5:   Find the point **p**_*k*_ on M closest to ${\text{p}}_{k}^{c}$ using barycentric coordinates Eq (2)

 6:  **end for**

 7:  Update the cage vertices **c**_*j*_ by solving the optimization Eq (3)

 8:  Update the mesh vertices using the new cage vertices and Eq (1)

 9: **end for**

## Result and discussion

To demonstrate the feasibility of our curve-based template deformation, we segmented three organs from swallowing 4DCT images. We also conducted a user study comparing our method with traditional FFD to evaluate the usability of the proposed method. For these experiments, we used three 4DCT images, which we refer to as CT_A, CT_B, and CT_C. These 4DCTs were obtained by capturing three volunteers using a 320-row area detector CT (Aquilion ONE, Canon Medical Systems). During the imaging, the volunteers swallowed thickened liquid containing a contrast agent. The imaging was conducted at a speed of 0.1 seconds per frame over approximately 2—3 seconds, resulting in approximately 20—30 frames. The voxel resolution of each frame of the 4DCTs is 512 × 512 × 320. The numbers of frames for the 4DCTs are as follows: 29 for CT_A, 25 for CT_B, and 20 for CT_C.

### Segmenting swallowing 4DCT volumes

**Fig 8:**
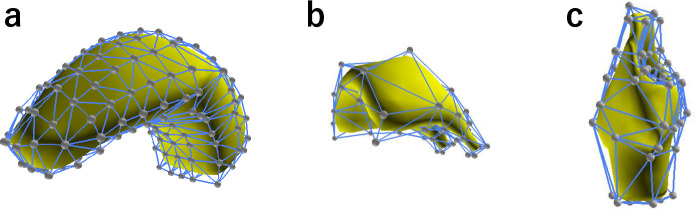
Template models for (a) tongue, (b) soft palate, and (c) larynx.

We performed segmentation of the three 4DCT images. In this experiment, we focused on organs strongly related to the swallowing motion, namely, the tongue, soft palate, and larynx. We prepared template models for each of them, as shown in Fig 8. We designed their cages such that they contain enough degrees of freedom to represent motion during swallowing.

**Fig 9:**
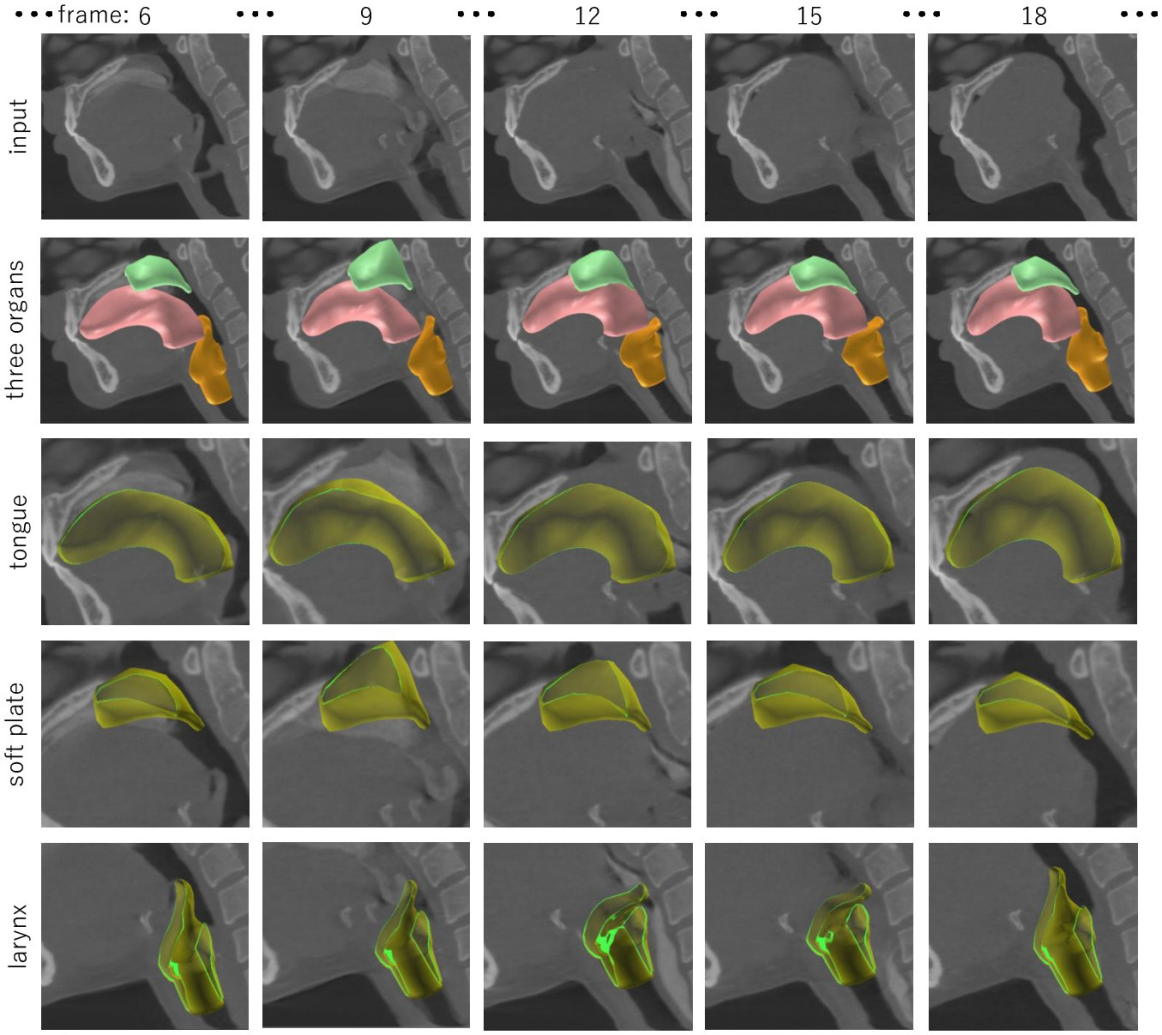
Segmentation result of 4DCT (CT_A) capturing a swallowing motion.

**Fig 10:**
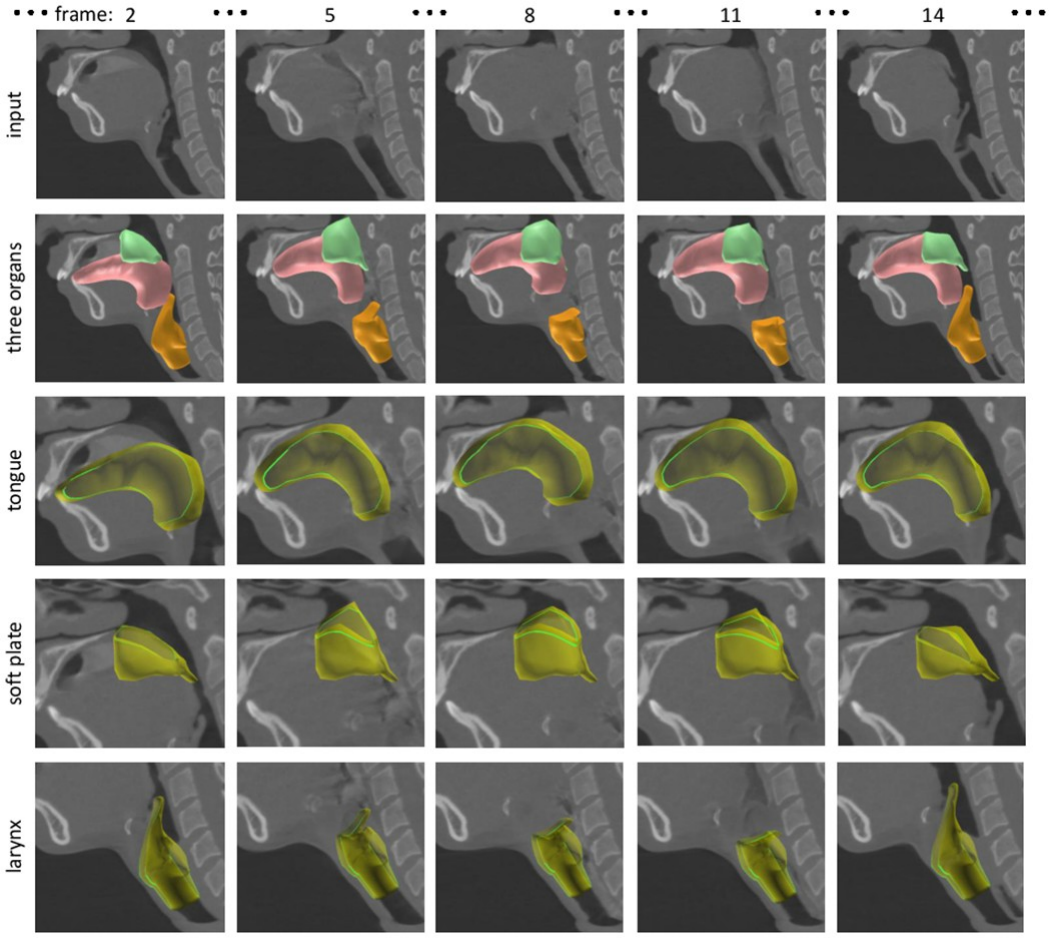
Segmentation result of 4DCT (CT_B) capturing a swallowing motion.

**Fig 11:**
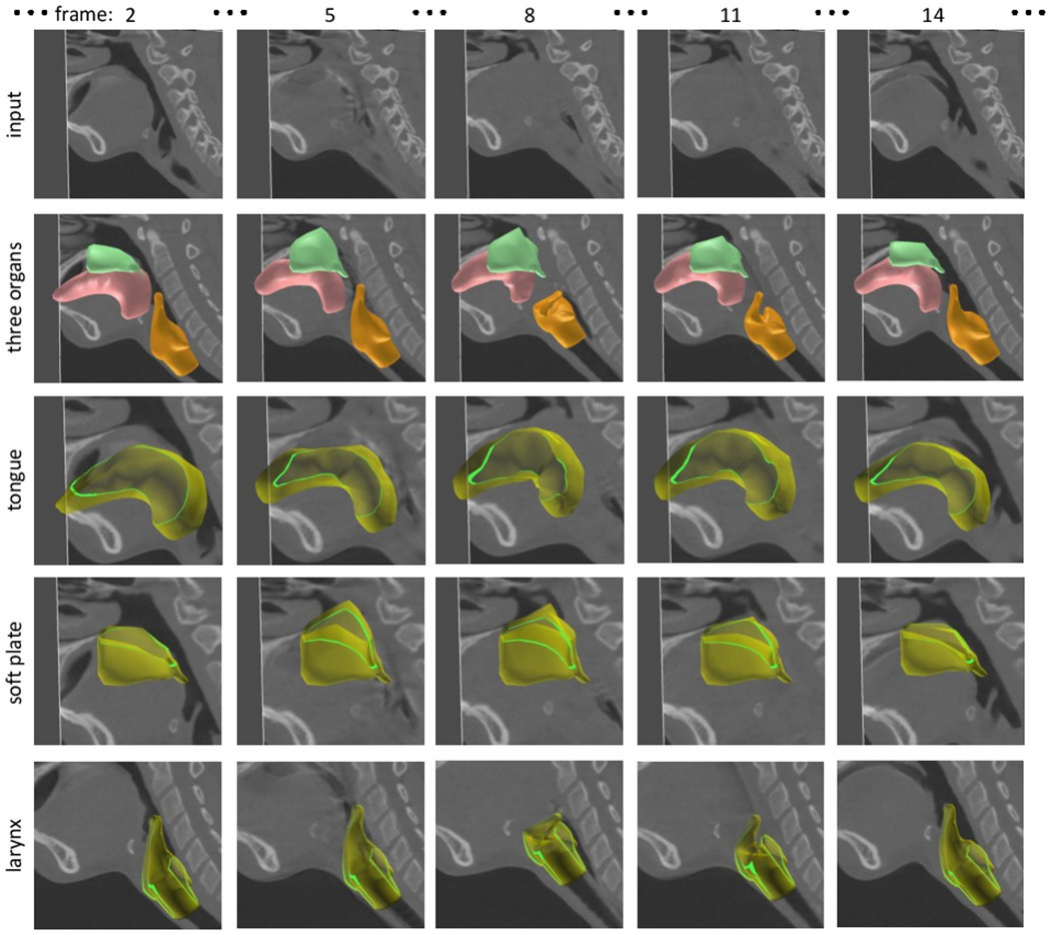
Segmentation result of 4DCT (CT_C) capturing a swallowing motion.

Figs 9–11 and S1 Video show the segmentation results for the three organs performed by the authors. From the segmentation result, we can observe the motion of the tongue smoothly transferring liquid toward the back of the throat. We can also observe that the motions of the soft palate and larynx are reproduced from the 4DCT; the soft palate moves backward to prevent the liquid from entering the nose, and the epiglottis of the larynx bends to prevent the liquid from going into the windpipe. These segmentation results were validated by the author group including a physician in the field of oral surgery.

Table 1 summarizes the interaction time and the number of curve constraints used for segmentation. In the case of CT_A segmentation, considering that 29 volumes were segmented for each organ, our method provided a highly efficient segmentation process for 4DCT; segmenting a single frame took approximately 5 min for the tongue, 3 min for the soft palate, and 5 min for the larynx. For the other two cases, our method achieved supporting similar efficient segmentation.

**Table 1:** Interaction time and the number (#) of curve constraints used in segmentation for three 4DCT images.

		tongue		soft palate		larynx	
4DCT	# frames	time	# curves	time	# curves	time	# curves
CT_A	29	137	261	87	87	135	191
CT_B	25	121	225	78	75	119	155
CT_C	20	98	180	67	60	96	127

**Fig 12:**
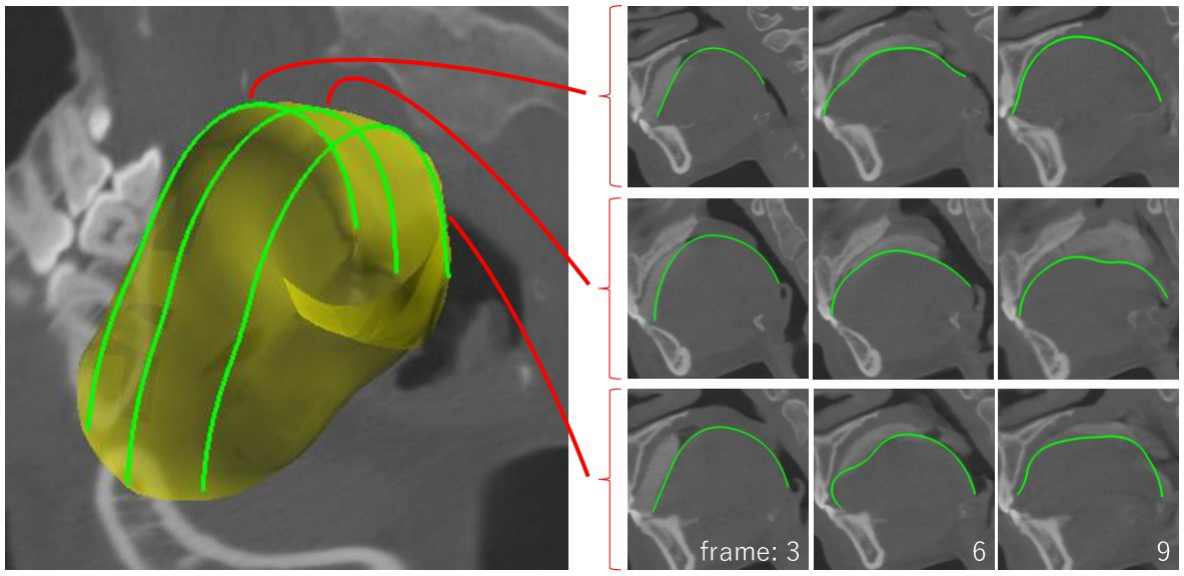
Shared curves placed on the tongue template.

In the segmentation of the soft palate and larynx, the cage-based FFD tool was also used. Specifically, it was used to fine-tune the shapes of the soft palate and roughly specify the bending motion of the epiglottis. As our method computes the deformation of the FFD cage by curve constraints, it has the advantage that the user can switch between the curve-based template deformation and cage-based FFD at any time. Moreover, in the segmentation of the tongue, the shared curve was used; three shared curves were placed at the boundary of the tongue on different sagittal planes (vertical cross-sections) where the organ appears clearly in all frames (Fig 12). Because the shapes of the shared curves are interpolated along time, the user needs to modify them only for the frames in which they do not match the target boundary of the 4DCT. This supports an efficient segmentation process.

### User study

To evaluate the usability of our method, we conducted a user study. The user study aims to confirm how efficiently users who have knowledge of the target region can deform templates using the proposed method and an existing cage-based FFD method. The participants were four undergraduate and graduate students. While one participant had an experience with 4DCT segmentation using the cage-based FFD, the others had no experience with 4DCT segmentation. The participants were engaged in a 15 min tutorial to learn the target region (i.e., tongue) in the swallowing 4DCT image and the usage of segmentation methods. They were then asked to segment the tongue in three representative frames using our curve-based method and the traditional cage-based FFD. They finished the task when they are satisfied with segmentation results. Since the tongue does not move largely before and after swallowing, we selected 3rd, 6th, and 9th frames of the 4DCT in Fig 2 where the swallowing motion begins and the tongue moves significantly. The order of the two methods was balanced among the participants. For each method, the participants roughly aligned the template by translating, rotating, and scaling it and then deformed it three times. We evaluated the time taken for segmentation by using the both methods.

Table 2 summarizes the time that each participant took for the initial alignment and deformation processes using the cage-based FFD and proposed curve-based method. All participants completed the deformation much faster using the curve-based method than the cage-based FFD. We observed that the interaction time for deformation was reduced by approximately 80% on average (five times faster). The proposed method allows the user to specify the boundary shape of the template directly by placing curves on the cross-sections, which we believe supports a more efficient segmentation process than the indirect cage-based FFD.

**Table 2:** Time [min] for aligning and deforming the template using the cage-based FFD and our curve-based methods. The alignments were performed once at the beginning with the same tool for each method. The deformation time represents the total time needed to deform the template for the three frames.

user	Cage-based		Curve-based (ours)	
	align	deform	align	deform
UserA	3	85	3	21
UserB	6	130	4	23
UserC	3	75	3	19
UserD	2	111	2	17

## Conclusion

This study presents an interactive method for segmenting organs from 4DCT images by deforming a template model to fit a target organ. The template is composed of a 3D mesh with an FFD cage enclosing the mesh. The user deforms the template by placing multiple curve constraints to specify its boundary shape. We also present a shared curve constraint applied to all frames and interpolated along the time axis. Our method formulates the template deformation using the FFD cage modification, allowing the user to switch between the curve-based deformation and traditional FFD at any time. To evaluate the feasibility of our method, we segmented three organs, such as the tongue, soft palate, and larynx, from a swallowing 4DCT image. Consequently, we were able to efficiently reconstruct the motions of these organs from 4DCT. We also conducted a user study to compare our curve-based method with the cage-based FFD. We found that our method could reduce the interaction time by approximately 20% compared to the traditional cage-based FFD method.

**Fig 13:**
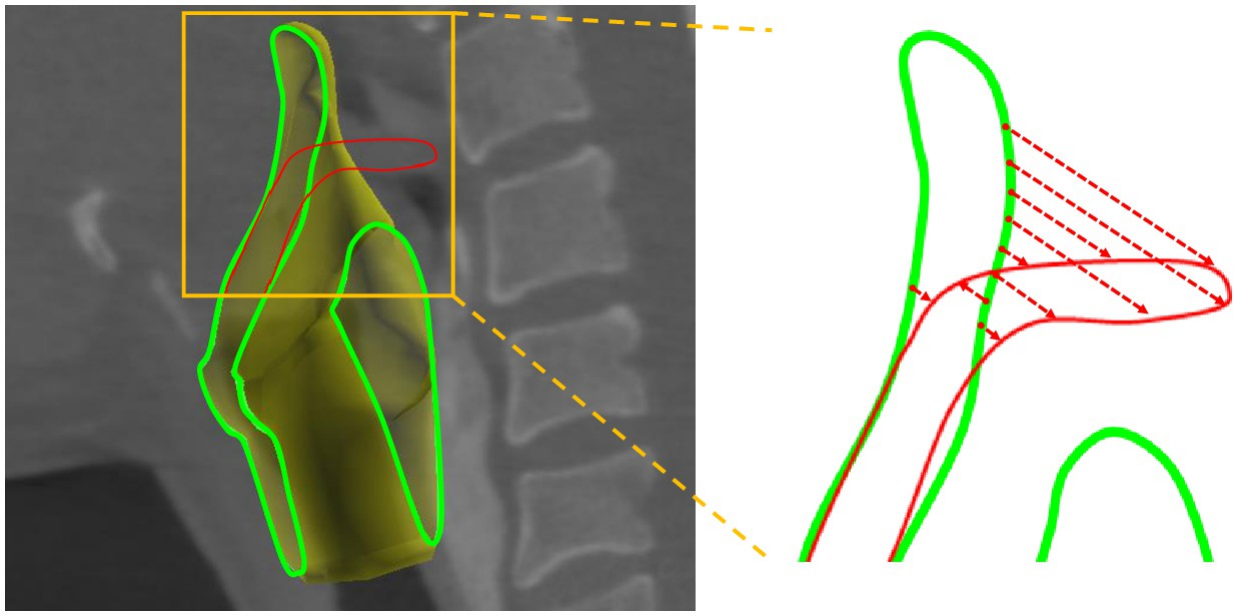
Limitation of the proposed method. When a constraint curve is significantly different from the shape of the template, our method could generate inaccurate associations.

One limitation of our method is that it is difficult to deform the template, such that its boundary shape significantly changes, only using curve constraints; for instance, globally bending the epiglottis larynx with curve constraints is difficult. This is because our method associates each constraint point with the closest point on the template mesh, and this association could be inaccurate when the current template shape is significantly different from constraint curves (Fig 13). One solution to this problem, which we used for larynx segmentation in Figs 9–11, is to perform a coarse deformation using the cage-based FFD before applying our curve constraints. Another solution is to improve the matching process between the curve constraints and boundary of the template, considering their shapes [36], which remains our future work. We also plan in the future to apply DNNs to support an even more efficient segmentation process. Using the dataset constructed with the proposed method, we would like to train a network to estimate the initial shapes of the templates.

## Supporting information

S1 Video Overview of the presented carve-based template deformation and segmentation results of the three 4DCT images. (MP4)
